# Numerical and Experimental Analysis of Mechanical Properties of Natural-Fiber-Reinforced Hybrid Polymer Composites and the Effect on Matrix Material

**DOI:** 10.3390/polym14132612

**Published:** 2022-06-27

**Authors:** Ayyappa Atmakuri, Arvydas Palevicius, Andrius Vilkauskas, Giedrius Janusas

**Affiliations:** Faculty of Mechanical Engineering and Design, Kaunas University of Technology, Studuntu 56, 51424 Kaunas, Lithuania; arvydas.palevicius@ktu.lt (A.P.); andrius.vilkauskas@ktu.lt (A.V.); giedrius.janusas@ktu.lt (G.J.)

**Keywords:** natural fibers, matrix material, mechanical properties, SEM-EDX, Halpin–Tsai model

## Abstract

The impact of matrix material on the mechanical properties of natural-fiber-reinforced hybrid composites was studied by comparing their experimental, and numerical analysis results. In the present work hemp and flax fibers were used as reinforcement and epoxy resin and ecopoxy resin along with hardener were used as matrix materials. To study the influence of the matrix material, two sets of hybrid composites were fabricated by varying the matrix material. The composite samples were fabricated by using the compression-molding technique followed by a hand layup process. A total of five different composites were fabricated by varying the weight fraction of fiber material in each set based on the rule of the hybridization process. After fabrication, the mechanical properties of the composite samples were tested and morphological studies were analyzed by using SEM-EDX analysis. The flexural-test fractured specimens were analyzed by using a scanning electron microscope (SEM). In addition, theoretical analysis of the elastic properties of hybrid composites was carried out by using the Halpin–Tsai approach. The results showed that the hybrid composites had superior properties to individual fiber composites. Overall, epoxy resin matrix composites exhibited superior properties to ecopoxy matrix composites.

## 1. Introduction

Natural fiber composites have gained much popularity in recent times due to concerns about the environmental impact of synthetic fiber composites [1,2]. Composites of natural fibers in thermosetting or thermoplastic polymer matrices have attracted a significant amount of interest from researchers due to their cost-effectiveness, ease of availability, environmental friendliness, and ability to compete with synthetic composites [3,4]. Natural-fiber-reinforced polymer composites have been employed in a variety of industries, including in automobile parts, aerospace components, sporting products, marine applications, military goods, and in the biomedical, microfluidics, and construction industries [5,6,7]. Low self-weight, high specific strength, free formability, and strong corrosion and fatigue resistance of natural-fiber-reinforced composites are some of the additional reasons for their increased popularity. On the other hand, natural composites have been limited in their usage due to their poorer mechanical and thermo-physical qualities when compared to synthetic composites and traditional structural materials [8,9]. Plant-based natural fibers such as banana, pineapple, abaca, jute, okra, sisal, hemp, flax, caryota, kenaf, and date palm fibers have proved to be potential reinforcement in the thermosetting and thermoplastic polymer composites and are being used in a wide range of applications [10,11].

In a composite material both the reinforcement and matrix materials play a crucial role. The reinforcement material acts as the load-carrier whereas the matrix material acts as the load-distributer when an external load is applied to the composite [12,13]. The matrix materials are classified as thermoset polymers and thermoplastic polymers. Thermoplastic materials can be reused even after the curing process, but need proper heat treatment to enable their reuse. Polyester, polypropylene, nylon, and Teflon are some examples of thermoplastic polymers. Unlike thermoplastic polymers, thermoset polymers cannot be used after the curing process. When the liquid thermoset resin is condensed (solidified) at a necessary temperature, it cannot recycle. The most common thermoset resins are epoxies, polyamides, and vinyl esters. In recent years, thermoset polymers have become more popular than thermoplastic polymers due to their wide range of applications in various sectors [14,15,16]. Among all polymer composites, thermoset polymers based on epoxy resin composites have had the most attention due to their good adhesive properties, low contraction, curing (liquid-state to solid-state) time, and ease of use [17,18].

The static and dynamic mechanical properties of the polymer composites reinforced with natural fibers are mostly the result of the quality and quantity of fiber materials [19,20], in addition to the interfacial strength and adhesion properties between the constituents of polymer composite [21]. Hybridization of composite materials is an alternative way to improve their mechanical performance. Hybridization occurs when a composite is produced by the combination of two or more reinforcements under the same matrix material, and the composites are called hybrid composites [22,23]. Several researchers have analyzed the mechanical properties of hybrid composites and shown the advancement in their properties. The main advantage of hybridization is that the mixing of the reinforcements can be done by various approaches such as blended continuous fibers, short fibers, discontinuous fibers, and particulate-matter fibers [24,25,26]. By far most of the hybrid compositions are focused on natural fibers with synthetic fibers such as glass and carbon fibers and they offered a range of properties superior to individual fiber composites. However, natural fiber hybrid composites have become more popular than synthetic fiber composites due to environmental concerns [27].

The chemical composition of natural fibers plays a prominent role in plant-based fiber composites, but environmental conditions such as climate dissimilarity and geographical changes influence the composition of natural fibers [28]. Hence, in most of the cases, their composition is expressed as maximum and minimum values [29]. In general plant-based fibers mainly consist of cellulose, hemicellulose, and lignin. Cellulose content and the aspect/ratio of natural fibers play a key role in mechanical performance [30,31]. In the absence of these chemical compositions in artificial (synthetic) fibers, their mechanical properties do not change much. On the other hand, natural fibers grow in open environments with the assistance of air, light, water, soil, etc. These conditions vary from time to time, which affects the properties of the fibers [32,33]. Some research studies have shown that fiber composites reinforced with cellulose-rich plant-based natural fibers such as hemp, flax, and banana have superior properties to composites reinforced with natural fibers with lower cellulose content [34]. Hence, in the current research hemp and flax fibers were used as a reinforcement to study the effect of matrix material on mechanical performance.

Recently, several researchers have shown an interest in working with natural fiber composites in the presence of thermoset polymers. Although there are many advantages with natural fiber composites the applications are still limited due to moisture uptake and flame-retardancy properties. Yihui et al. [35] developed a micromechanical model to understand the effect of moisture on the degradation of composite material. The mechanical degradation and swelling properties of natural fiber composites were analyzed by the Mori–Tanaka model with the inclusion of different shapes. The results showed that a proper matrix material can avoid mechanical degradation as well as reduce the moisture uptake by the natural fiber composite. Neves et al. [36] investigated the mechanical properties of hemp fiber composites with various matrix materials. They drew a comparison between epoxy and polyester resins. They showed that hemp fiber composites based on epoxy resin polymer have superior properties to polyester resin composites and stated that these composites have a potential uses in armed applications. Sapuan et al. [37] investigated the mechanical properties of woven banana-fiber-reinforced epoxy composites. A theoretical model based on the ANOVA technique was developed to compare the experimental results and it was observed that there was a slight variation in the theoretical and experimental results. Several researchers have offered useful and comprehensive review articles on natural-fiber-reinforced hybrid composites and the importance in terms of mechanical performance [10,38,39]. Summerscales et al. [40] reviewed various statistical models which have been useful for natural fiber composites. This paper also presents the various harvesting techniques and growing conditions of natural fibers. Alhazmi et al. [41] worked on tribological and mechanical properties of hybrid composites based on epoxy resin. The results showed that the presence of nanofiller materials cannot affect any sort of improvement in terms of wear resistance of the composite material. Singh et al. [42] investigated the mechanical properties of hemp fiber composites. To analyze the mechanical properties, tensile and flexural tests were conducted. The results showed that the mechanical properties were increased with an increase in fiber content. These properties increased up to 40% and then decreased. Moudood et al. [43] studied the mechanical properties of flax fibers under various environmental conditions. The mechanical properties that were measured experimentally agreed strongly with the statistical results.

Zhong et al. [44] reviewed the challenges in numerical simulations and modeling of natural fiber composites. Microstructures of natural fiber composites were presented by featuring their properties that should be viewed before displaying. The authors also discussed and summarized the previous numerical works. Unlike artificial fibers, natural fiber composites certainly stand out concerning numerical approaches [45]. Existing specialized difficulties in this subject are summed up to give possible options for future research. Jose da Silva et al. [46] worked on numerical and experimental analysis of natural-fiber composites. This work primarily emphasized sisal and banana natural-fiber-reinforced epoxy-resin polymer composites. Tensile tests were performed to validate the numerical data. To verify the experimental results both 3D and 2D models were developed by considering the elastic properties of each constitution of the composite material. Two-dimensional analysis was conducted based on the rule of mixtures (ROM) and three-dimensional analysis was conducted on ANSYS software. The results stated that the variation of Poisson’s ratio values did not affect the finite element analysis results. Sathishkumar et al. [47] studied the experimental and numerical analysis of filler material on sisal-fiber-reinforced polymer composites. The mechanical properties were tested by using flexural and tensile tests. The 2D analysis of polymer composites was conducted by using the ROM method and Halpin–Tsai approach. The 3D analysis was conducted by using the ANSYS workbench by considering the elastic properties of the materials used for fabrication purposes. The results showed the improvement in mechanical properties due to the addition of filler material to the composite materials. Prasanthi et al. [48] investigated the waviness effect on sisal fiber composites through numerical and experimental methods. The idea of the work was to understand the effect of waviness on fiber composites and compare the results in both analytical and experimental methods. The analytical works were carried out by using ANSYS model and the results stated that there was a significant effect of waviness on the fiber composites. Balasubramanian et al. [49] analyzed the mechanical properties of natural fibers by using experimental and FEA analysis. Hybrid composites were fabricated by using flax, sisal, and aloe vera fibers as reinforcement and epoxy resin as a matrix material. The mechanical properties were tested by using tensile, flexural, and impact tests. The finite element analysis was carried out to find the stress, deformation, and displacement of the composite materials, and then the results were compared.

From the above literature, two significant points are perceived. One is that the mechanical properties of natural fibers can be improved by using a hybridization process and the second is that the numerical and theoretical analysis of natural fibers can be done by assuming definite properties through finite element analysis and micromechanics. With this this information, the main objective of our research on the effect of matrix materials on natural fiber hybrid composites was defined. This objective was to understand the impact of matrix material on the mechanical properties of the natural-fiber-reinforced epoxy and ecopoxy hybrid composites. For this study, hemp and flax fibers were used as reinforcements, and to draw the contrast between matrix materials, epoxy and ecopoxy materials were used as matrix materials. The results are assessed by using experimental and numerical methods. The mechanical properties of hybrid composites were tested by using flexural and interlaminar shear properties. The wettability properties are tested by using contact angle measurement and the morphological studies were carried out with SEM-EDX spectrum analysis. Theoretical analysis of composite materials was estimated by using Halpin–Tsai method and the effect of matrix material on mechanical properties of natural fibers hybrid composites was addressed.

## 2. Materials and Methods

### 2.1. Materials

To carry out the current research work, hemp and flax fibers were procured from Naturalus Pluostas UAB, Vilnius, Lithuania. The epoxy resin and its hardener were procured from Composite 24, Riga, Latvia and the ecopoxy (the bio-based resin contained 36% of bioactivity) resin along with hardener was procured from EcoPoxy EU. Warsaw, Poland. Teak wood was used for the preparation of mold material.

### 2.2. Preparation of Reinforcement and Matrix Materials

The reinforcement materials were available in the form of a bundle. Each fiber was carefully chosen by eliminating the unwanted materials, such as the thick stems and small pellets by using the hand sitting process. Once the waste materials had been separated from the fibers, water treatment was carried out and the fibers were dried until the moisture was eliminated. However, the fibers were still not ready for fabrication due to their smooth surface finish and poor adhesive properties. Therefore the separated fibers underwent a chemical treatment process. The fibers were treated with a 5% NaOH solution and then cleaned with distilled water. After alkaline treatment, the fibers were dried in an oven ready for the fabrication process.

The matrix materials used for the fabrication process were epoxy and ecopoxy resin along with hardener material. The weight portion of both the matrices was considered a 2:1 ratio as per the material description provided by the company from which they were procured. The required amount of resin and hardener were put into a plastic container and stirred with a plastic stirrer for 4 minutes and the walls of the container were scratched properly to get the proper mix and left for two minutes to allow the clear solution to settle. The process was repeated for both matrix materials. Once the mixed solution had settled it was taken for the fabrication process.

### 2.3. Composite Sample Preparation

**Figure 1:**
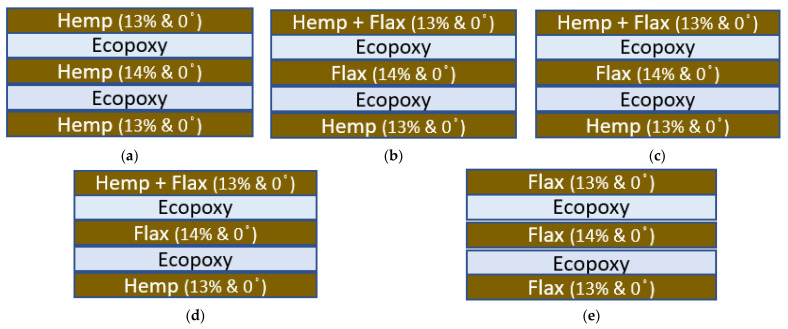
Schematic layout of the ply sequence for ecopoxy-matrix-based hybrid composites. (**a**) 40H/0F, (**b**) 25H/15F, (**c**) 20H/20F, (**d**) 15H/25F, and (**e**) 0H/40F.

The weight fractions of the reinforcement and matrix materials were considered as per the rule of the hybridization process. This states that a composite sample is fabricated by using two or more fiber laminates under the same resin material with a 0.4 Wf.% [50,51]. In the present research work, hemp (H) and flax (F) fiber hybrid composites were fabricated in the presence of epoxy resin and ecopoxy resin. The composites were fabricated by using the compression molding technique followed by the hand-layup technique. The required weight proportions of fibers were placed uniformly in the mold material and compressed to acquire the mold shape and then the epoxy resin was applied to it. The fibers were placed in uniform direction (“0” degrees) for neat hemp and flax composites whereas in the hybrid case the fibers were placed in uniform-direction layer-by-layer mode and for testing the orientation of fibers was not considered. The schematic representation of the ply sequence for ecopoxy composites is given in the Figure 1 and a similar sequence is followed for the epoxy-matrix-based hybrid composites. The mold material was covered with a backing paper and then compressed with a uniform load for 72 hours. Once the curing process was completed the composites were subjected to the heat treatment process. A total of five different weight fractions of composites were fabricated (40H/0F, 25H/15F, 20H/20F,15H/25F, and 0H/40F). In all cases the fibers were laid down horizontally with a 0 degree angle. The sample weight fractions are given in Table 1. All the samples were fabricated as per ASTM testing standards. The same process was repeated for ecopoxy matrix composites.

### 2.4. Characterization Methods

#### 2.4.1. Flexural Test

To measure the flexural properties such as flexural strength and modulus of the composite samples, a universal testing machine (Tinus Olsen H10K) was used. To test the samples, the 3-point bending test was preferred over the 4-point bending test because the external load acts at the center of the composite so load distribution was uniform [52]. The test was conducted at a constant crosshead speed of 10 mm/min and the strain rate was 0.10 mm/min for all specimens. All the samples were tested as per standard ASTM D-790 [53]. For each sample, a total of five composites was tested to take average values. The flexural modulus of a composite sample was calculated by drawing a tangent line for the stress–strain curve; hence it is also called the tangent modulus. In general, the flexural modulus is defined as the capacity of a composite sample to deform when an external load acts on it. The flexural test samples were analyzed by using the scanning electron microscope.

#### 2.4.2. Interlaminar Shear Test

To calculate the interlaminar shear strength of composite material, a universal testing machine with a full load capacity of 10 KN was used. To test the samples, the composite was fixed on both ends in between the jaws of the testing machine, and the load was applied in an axial direction from one end and the lower end was fixed. The samples were tested as per standard ASTM D-3846 [54]. The rectangular samples had a notch cut on both sides, 6.4 mm apart. The notch depth was considered half of the thickness of the sample on each side of the composites specimen. The shear strength was measured only when failure occurred between the notches. The load acting on the composite was gradually increased until the composite broke.

#### 2.4.3. Wettability Test

The wettability properties of composite material were tested by using the contact angle measurement process. This provides quantitative information on interfacial energy between a fluid drop and composite surface [55]. A variety of fluids, such as spirit, water, and glycerin are used to measure the contact angle of a solid surface, of which, water is the most popular. Wettability indicates the examination of contact angles that regulate the wetting degree between a solid surface and a liquid drop. If the contact angle is less than 90 degrees it means the solid surface has a hydrophilic nature and if it is more than 90 degrees it has a hydrophobic nature. There are several factors that can affect the contact angle such as temperature, atmospheric conditions, type of liquid, the viscosity of the liquid, and time to take the measurement [56,57]. In the present work, all the samples were tested in a dark room to avoid light, and dimensions were considered as per ASTM testing standards. The composite surfaces were cleaned properly before forming the water bubble. The contact angle values were measured at various positions on the composite surface.

#### 2.4.4. SEM Energy Dispersive X-ray Spectrum Analysis

A scanning electron microscope (SEM) from Bruker, Billerica, MA, USA was used to investigate the morphology of both the composite surface and the fractured sample. The element composition the surface of each composite sample and the facture position were evaluated by using SEM-EDX spectroscopy. The error % connected with the elemental composition analysis was evaluated.

### 2.5. Numerical Analysis

Numerical analysis of natural fiber composites was carried out by the using micromechanical method i.e., the Halpin–Tsai approach. Although there are many methods available to predict theoretical analysis, these are easy to apply to natural fiber composites. To calculate the elastic properties the basic assumptions must be considered as the composite sample was isotropic and homogeneous, as well as being linear elastic in nature [58,59]. We modified the equations to analyze the hybrid fiber composite elastic properties. The modified equations to calculate the elastic properties are given below:

Halpin–Tsai method [60,61,62]

(1) $$E=\frac{E_{m}\left(1+\zeta \eta V_{f}\right)}{1-\eta V_{f}}$$

where *η* is

(2) $$\eta =\frac{E_{f}-E_{m}}{E_{f}+\zeta E_{m}}$$

By substituting the *η* value in Equation (3) the final equation will be as follows:

(3) $$E=\frac{E_{m}+\zeta V_{f}E_{m}\left(E_{f}-E_{m}\right)}{E_{f}+\zeta E_{m}-V_{f}\left(E_{f}-E_{m}\right)}$$

where *E_f_* and *E_m_* are the Young’s modulus of fiber and matrix material, respectively. *V_f_* and *V_m_* are volume fractions of fiber and matrix material, respectively. *ζ* is an arbitrary variable, also called an empirical factor considered based on the assumptions. The *ζ* values are taken as 0, 1, 2, and so on. The modified Halpin’s equation is as follows:

(4) $$E\;=\frac{E_{m}+\zeta E_{m}(V_{f1}+V_{f2})\left(E_{f1}+E_{f2}-E_{m}\right)}{E_{f1}+E_{f2}+\zeta E_{m}-(V_{f1}+V_{f2})\left(E_{f1}+E_{f2}-E_{m}\right)}$$

*E_f_*_1_, *E_f_*_2_, and *E_m_* are Young’s modulus of hemp fiber and flax fiber, and the matrix material, respectively. *V_f_*_1_, *V_f_*_2_, and *V_m_* are the volume fractions of hemp fiber, flax fiber, and matrix material, respectively. In the present work, an attempt has been made to check the extent of the modified Halpin’s equation to the natural fiber hybrid composites. The test was conducted by assigning properties such as Poisson’s ratio, young’s modulus, density, bulk modulus, and shear modulus to the material. The values are given in Table 2. The mechanical properties such as elastic modulus and displacement were determined and the results were compared with the theoretical and experimental results.

## 3. Results

### 3.1. Flexural Properties

**Figure 2:**
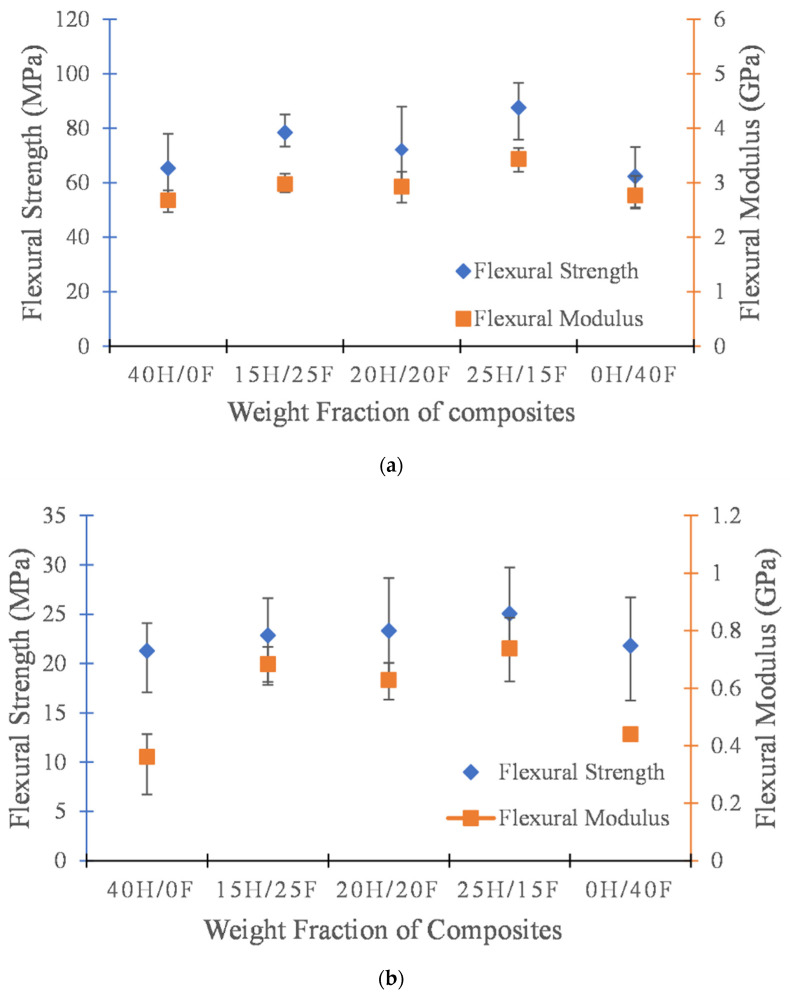
Flexural strength and modulus of H/F hybrid composites with epoxy (**a**) and Ecopoxy (**b**) matrices.

The flexural strength and flexural modulus of hemp and flax hybrid composites with various weight fractions in the presence of epoxy resin matrix and ecopoxy resin matrix are shown in Figure 2. In both cases, hybrid composites showed superior flexural properties to those of individual fiber composites. This reflects the impact of hybridization on composite materials. It was observed that the epoxy-matrix-based composites exhibited greater flexural strength and flexural modulus than the ecopoxy matrix composites. This can be attributed to poor bonding and adhesion properties between the ecopoxy matrix and the reinforcement material. The epoxy matrix composites 25H/15F exhibited the highest flexural strength and modulus and the lowest was observed in the 0h/40F composites, whereas in ecopoxy matrix composites, 25H/15F hybrid composites showed the highest values and 40H/0F composites showed the lowest values. These results proved that the matrix material plays a crucial role in the mechanical performance of the composite material.

**Figure 3:**
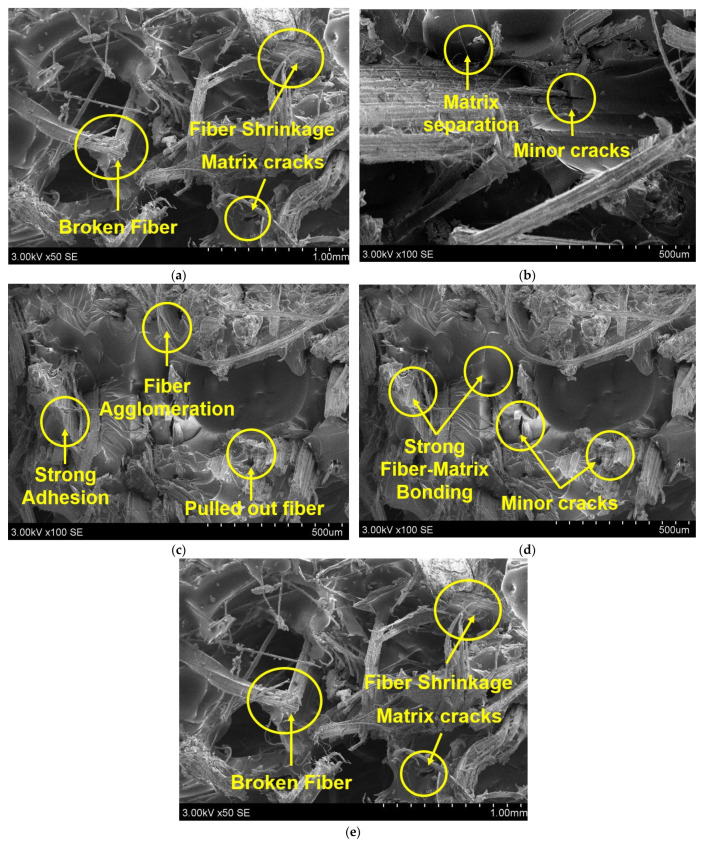
SEM analysis of flexural tested hemp- and flax-fiber-reinforced epoxy matrix composites. (**a**) 40H/0F, (**b**) 15H/25F, (**c**) 20H/20F, (**d**) 25H/15F, and (**e**) 0H/40F.

Scanning electron microscope analysis of flexural test specimens of epoxy matrix composites is illustrated in Figure 2. The presence of broken of fibers and fiber shrinkage in individual fiber composites can be observed (Figure 3a,e). This lowers the strength of the composite, whereas in hybrid composites (Figure 3b–d) strong bonding between reinforcement and matrix material was observed. This strong adhesion property of composites improves the stress distribution inside the composite material and improves the strength of a composite. In addition, minor cracks were observed in hybrid composites.

### 3.2. Interlaminar Shear Properties

**Figure 4:**
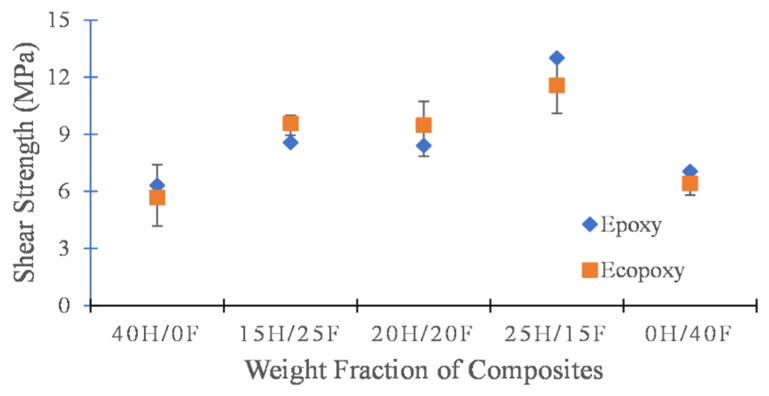
Interlaminar shear strength of epoxy and ecopoxy composites with various weight fractions.

The experimental results of epoxy and ecopoxy-based hybrid composites obtained from interlaminar shear tests are presented in Figure 4. The shear strength of a composite is one of the crucial parameters in defining the capability of a composite to withstand delamination damage. From the results, it was clear that the interlaminar shear strength properties of hybrid composites were superior to those of the pure fiber (40H/0F and 0H/40F) composites in both cases. This indicates the strong bonding properties between the layers of the hybrid composite compared to the pure composite. The epoxy-based 25H/15F hybrid composite showed the highest shear strength at 13.01 MPa and the ecopoxy-based 40H/0F composite exhibited the lowest at 5.67 MPa.

### 3.3. Wettability Analysis

**Figure 5:**
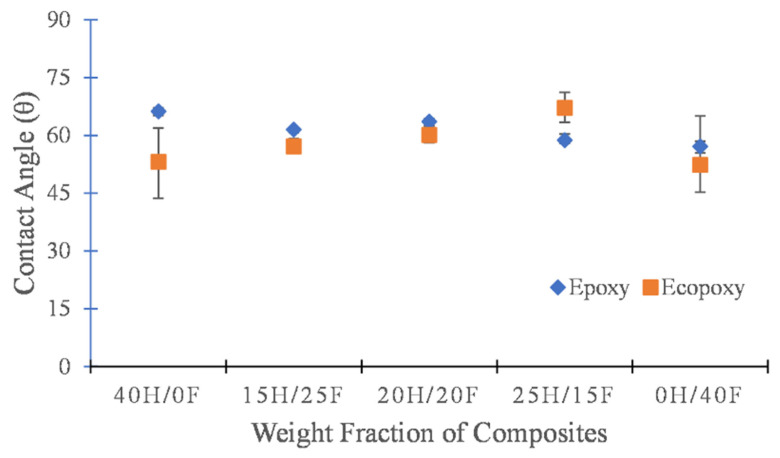
Contact angle measurement of epoxy and ecopoxy composites with various weight fractions.

To analyze the wettability properties of a composite material a contact angle measurement was conducted on the hemp and flax fiber composites and the results are illustrated in Figure 5. To understand the surface property of composite material, it is necessary to find the wetting nature of a composite [63]. It is easy to determine whether a composite is exhibiting a hydrophilic or a hydrophobic surface property by conducting the contact angle measurement analysis. It was found that both epoxy and ecopoxy composites showed contact angles of less than 90 degrees which meant that all composites exhibited hydrophilic surface nature. In both cases, hybrid composites showed a similar contact angle whereas pure composites exhibited a slight variation in it.

### 3.4. SEM-EDX Analysis

**Figure 6:**
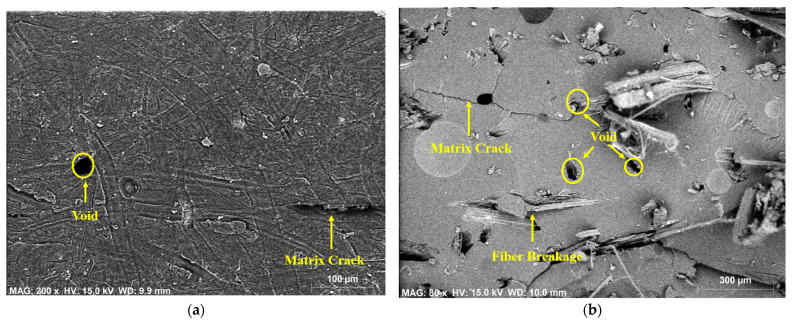
SEM EDX analysis on hemp-fiber-reinforced epoxy composite at surface (**a**) and fracture (**b**) positions.

**Figure 7:**
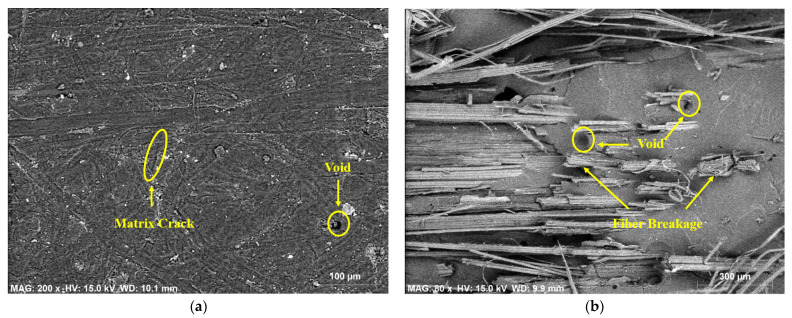
SEM EDX analysis of flax fiber reinforced epoxy composite at surface (**a**) and fracture (**b**) positions.

Morphological studies on individual hemp- and individual flax-fiber-reinforced epoxy composites at the surface position and the fracture position were carried out by using the scanning electron microscope energy dispersive X-ray spectroscopy and the results are shown in Figure 6 and Figure 7. From SEM results it was observed that the presence of defects such as void content, fiber breakage, and matrix cracks were greater at the fracture position than at the surface position. The reasons for this could be poor reinforcement–matrix interactions and agglomeration of the fibers in the composite material.

To determine the chemical composition and element analysis of the composite material, EDX spectrum analysis is usually preferred. The chemical composition and error % of the hemp and flax fiber epoxy composites are given in Table 3. In both fiber composites, carbon had the highest percentage followed by oxygen and the percentage of carbon was greater at the surface than at the fracture position. However, the atomic% of oxygen was greater at the fracture position than at the surface position. Only carbon, oxygen, chlorine, and a small amount of sodium were observed at the surface position, but at fracture position, calcium, aluminum, and potassium were observed in addition to those chemical compositions present at the surface of the epoxy-based composite material.

**Figure 8:**
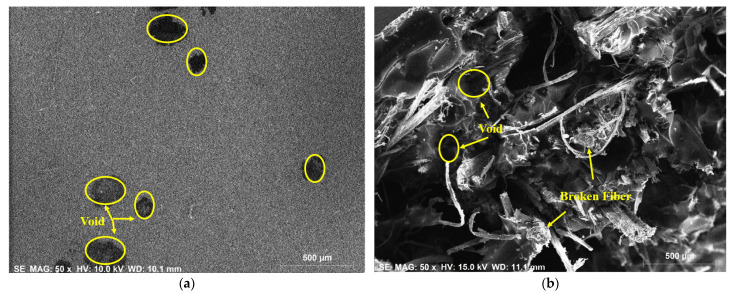
SEM EDX analysis on hemp fiber reinforced ecopoxy composite at surface (**a**) and fracture (**b**) positions.

**Figure 9:**
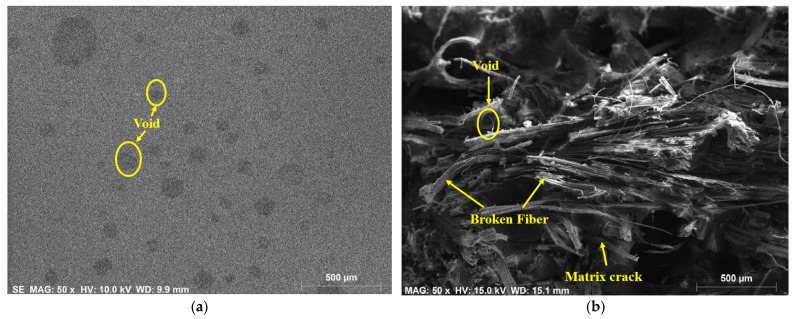
SEM EDX analysis on flax fiber reinforced ecopoxy composite at surface (**a**) and fracture (**b**) positions.

The morphological studies on individual hemp- and individual flax-fiber-reinforced ecopoxy matrix composites at the surface position and fracture position were carried out by using SEM-EDX spectroscopy and the results are shown in Figure 8 and Figure 9. From SEM results it was observed that, similarly to epoxy composites, the presence of defects such as void content, fiber breakage, and matrix cracks was greater at the fracture position than at the surface position. There were, however, more defects in the ecopoxy composites than in the epoxy composites. The presence of defects leads to the weakening of the composite strength. It ultimately affects the mechanical performance of the composite material.

The chemical composition and error % of the hemp and flax fiber ecopoxy composites are given in Table 4. In both fiber composites, oxygen had the highest percentage followed by carbon, The percentage of oxygen was higher at the fracture than at the surface position, whereas the atomic% of carbon was more at the surface position than the fracture position. Only carbon, oxygen, chlorine, and a small amount of silicon were observed at the surface position, but at the fracture position, carbon, oxygen, chlorine, silicon, and calcium were observed.

Overall, the defect percentage was greater in the ecopoxy composites than in the epoxy composites; hence, the strength of the composite was decreased in the ecopoxy case. Due to the biological nature of the ecopoxy resin material the percentage of oxygen was greater in ecopoxy composites than in the epoxy composites.

### 3.5. Numerical Analysis

**Figure 10:**
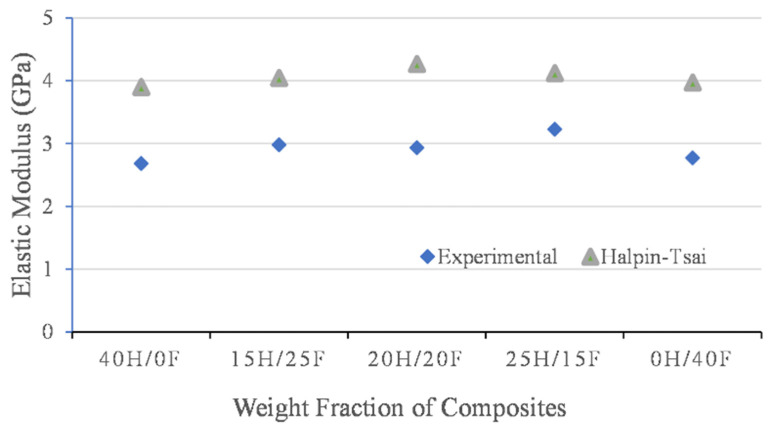
Elastic properties of epoxy-based hybrid composites by various methods.

**Figure 11:**
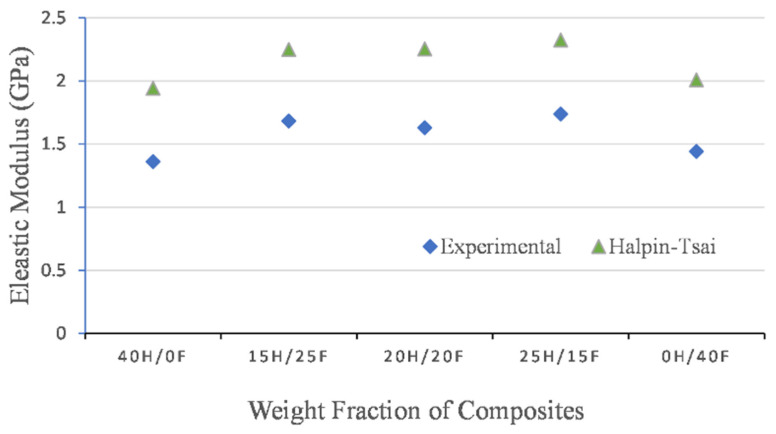
Elastic properties of ecopoxy-based hybrid composites by various methods.

Numerical analysis was carried out by using the modified Halpin’s method. The values are presented in Figure 10 and Figure 11. In both cases, experimental values and numerical values were not close to each other and a large variation was observed when using Halpin’s method. Halpin’s method gave the higher data for all the hybrid composites investigated. This means that the modified Halpin’s equations for calculation of elastic properties of hybrid-fiber-reinforced epoxy and ecopoxy composites gave higher bound results. Individual fiber composites showed the lowest elastic properties in both cases.

The data presented in Table 5 and Table 6 shows the % error calculated for the epoxy and ecopoxy-matrix-based hybrid fiber composite’s elastic properties. The error % was calculated by taking the ratio of the difference between theoretical values and experimental results upon the experimental results. It was observed that the error % calculated by using Halpin’s method was far higher than the other methods. This indicates that the modified Halpin–Tsai theoretical calculations cannot be applied to natural fiber hybrid composites. It was observed that the elastic properties obtained from the numerical models were slightly higher than the experimental results in both epoxy and ecopoxy cases. This can be attributed to the fabrication errors and environmental conditions during the experimentation process whereas in theoretical predication, the outside factors are not considered.

The percentage error was calculated for epoxy and ecopoxy hybrid composites. The error was calculated by taking the ratio of the difference between numerical values and experimental results. The maximum error % of elastic properties was observed in pure composites such as 0H/40F and 40H/0F in both epoxy and ecopoxy hybrid composites and the lowest was observed in 25H/15F epoxy hybrid composites. In the case of hybrid composites, the error % was similar whereas in pure composites it was different.

## 4. Discussion

Most of the previous research work has have been carried out on natural fiber reinforced thermosetting or thermoplastic hybrid composites. Natural fibers such as banana, okra, jute, bamboo, and pineapple were used as a constituent of composite material [64,65,66]. The present research was mainly focused on the impact of matrix material on the mechanical properties of a composite material. To draw a contrast between matrix materials, epoxy and ecopoxy (bio-based resin) were used as a matrix material.

The hemp- and flax-fiber-reinforced epoxy composites showed superior properties to the ecopoxy hybrid composites. In addition, hybrid composites exhibited superior properties to individual fiber composites. The results showed that epoxy-based hybrid composites had flexural strength properties ranging from 58 MPa to 80 MPa whereas in the previous research this was between 45 MPa to 64 MPa. Similarly, there was also a considerable development in the interlaminar shear properties. From contact angle measurement analysis, previous results showed that all the natural fiber composites exhibited hydrophilic surface properties (contact angle less than 70 degrees). However, in the current research, ecopoxy hybrid composites showed a contact angle close to 90 degrees which means that composites tend to show hydrophobic surface properties.

## 5. Conclusions

In the present research work, the effect of matrix material on the mechanical properties of composite material was investigated by drawing a comparison between the epoxy-resin-matrix natural fiber composites and ecopoxy (bio-resin)-resin-matrix natural hybrid fiber composites. The results are given below.

Epoxy-matrix-based hybrid composites showed superior mechanical properties to ecopoxy-matrix-based hybrid composites. It was observed that the mechanical properties of epoxy-resin hybrid composites were almost two times greater than ecopoxy composites. In both cases, pure composites showed the poorest mechanical properties such as flexural strength and interlaminar shear strength and hybrid composites exhibited the maximum strength and shear properties. In the case of epoxy composites, the maximum flexural and shear strength were observed as 78 MPa and 13MPa, respectively, whereas in the case of ecopoxy composites, values were 30MPa and 10 MPa, respectively. The wettability properties of natural fiber hybrid composites were investigated by conducting contact angle measurement. The results showed that all the composites showed hydrophilic (θ < 90˚) surface properties. The morphological studies and chemical composition presented in the composites were investigated by using SEM-RDX spectrum analysis.

To validate this research work, a numerical analysis was carried out including the experimental analysis. Theoretical studies were carried out to determine the elastic properties based on the modified Halpin–Tsai method. It was observed that the error % was less with the numerical methods but in the case of Halpin’s method, it was more. Hence Halpin’s method is not suitable for predicting the elastic properties of natural fiber polymer composites.

Overall epoxy-based natural hybrid composites showed improved mechanical properties compared to ecopoxy-based hybrid composites. Hence the hemp and flax fiber reinforced epoxy matrix composites could be a potential replacement for synthetic fiber composites in various industries such as the automobile, construction, microfluidics, and biomedical industries.

## Figures and Tables

**Table 1:** The weight fraction of composite samples for epoxy/ecopoxy matrices.

Composites	Hemp (%)	Flax (%)	Total Reinforcement Vol. (%)	Total Matrix Vol. (%)
40H/0F	40	0	40	60
25H/15F	25	15	40	60
20H/20F	20	20	40	60
15H/25F	15	25	40	60
0H/40F	0	40	40	60

**Table 2:** Mechanical properties of the materials.

Material	Density (g/cm^3^)	Poisson’s Ratio	Young’s Modulus (GPa)	Shear Modulus (GPa)	Bulk Modulus (GPa)
Epoxy resin	1.10	0.29	3.35	2.10	1.408
Flax	1.4–1.5	0.41	60	21.27	1.11
Hemp	1.48	0.39	70	25.18	1.06
Ecopoxy	1.13	0.23	2.28	0.98	1.26

**Table 3:** Chemical composition of hemp and flax fiber epoxy composites.

Element	Hemp Surface		Hemp Fracture		Flax Surface		Flax Fracture	
	Atom. C (%)	Error (%)	Atom. C (%)	Error (%)	Atom. C (%)	Error (%)	Atom. C (%)	Error (%)
Carbon	73.74	21.6	69.97	20.3	74.66	21.9	59.98	21.8
Oxygen	26.15	10.4	29.76	11.4	25.09	10.0	39.57	14.7
Chlorine	0.11	0.0	0.10	0.0	0.15	0.0	0.18	0.0
Calcium	--	--	0.09	0.0	--	--	0.14	0.0
Aluminum	--	--	0.08	0.0	--	--	--	--
Sodium	--	--	--	--	0.10	0.0	--	--
Potassium	--	--	--	--	--	--	0.13	0.0

**Table 4:** Chemical composition of hemp and flax fiber ecopoxy composites.

Element	Hemp Surface		Hemp Fracture		Flax Surface		Flax Fracture	
	Atom. C (%)	Error (%)	Atom. C (%)	Error (%)	Atom. C (%)	Error (%)	Atom. C (%)	Error (%)
Carbon	48.11	23.6	40.76	16.8	48.76	25.2	37.42	35.0
Oxygen	51.30	20.6	58.97	20.3	51.07	19.6	62.12	22.7
Chlorine	0.39	0.1	0.17	0.0	0.17	0.1	0.27	0.1
Silicon	0.20	0.1	--	--	--	--	--	--
Calcium	--	--	0.10	0.0	--	--	0.19	0.1

**Table 5:** Comparison of elastic properties of epoxy composites.

Epoxy Composites	Experimental	Halpin–Tsai Method	Error (%)
40H/0F	2.683	3.896	45.21
15H/25F	2.981	4.042	35.59
20H/20F	2.934	4.264	45.33
25H/15F	3.226	4.118	27.65
0H/40F	2.772	3.968	43.15

**Table 6:** Comparison of elastic properties of ecopoxy composites.

Ecopoxy Composites	Experimental	Halpin–Tsai Method	Error (%)
40H/0F	1.361	1.942	42.69
15H/25F	1.683	2.248	33.57
20H/20F	1.628	2.254	38.45
25H/15F	1.737	2.324	33.79
0H/40F	1.442	2.008	39.25

## Data Availability

Data sharing is not applicable.
